# The effect of timing of iron supplementation on iron absorption and haemoglobin in post-malaria anaemia: a longitudinal stable isotope study in Malawian toddlers

**DOI:** 10.1186/1475-2875-13-397

**Published:** 2014-10-10

**Authors:** Dominik Glinz, Moses Kamiyango, Kamija S Phiri, Francis Munthali, Christophe Zeder, Michael B Zimmermann, Richard F Hurrell, Rita Wegmüller

**Affiliations:** From the Laboratory of Human Nutrition, Institute of Food, Nutrition and Health, ETH Zürich, Schmelzbergstrasse 7, LFV CH-8092 Zurich, Switzerland; Basic Medical Sciences Department, College of Medicine, Blantyre 3, Malawi; Public Health Department, College of Medicine, Blantyre 3, Malawi; Health Centre Zomba, Zomba, Malawi

**Keywords:** Anaemia, Iron supplementation, Toddlers, Malaria, *Plasmodium falciparum*, Stable iron isotope, Haemoglobin, Malawi, Inflammation

## Abstract

**Background:**

In sub-Saharan Africa, children with *Plasmodium falciparum* malaria and anaemia are often given iron supplementation at the time of malaria treatment. Inflammation during and after malaria may decrease iron absorption, thus, absorption might be improved if the start of supplementation is delayed. The study objective was to measure iron absorption from iron supplements started immediately or delayed by two weeks after completion of therapy against uncomplicated *P. falciparum* malaria.

**Methods:**

Malawian toddlers (n = 48; age 12–24 months) were alternatively assigned to two groups according to their appearance at the health centre: group A was provided iron supplements (30 mg Fe daily) as a FeSO_4_-containing syrup for eight weeks starting immediately after malarial treatment; group B was given the iron after a two-week delay. Iron absorption from the syrup was measured on the first day of iron supplementation, and after two and eight weeks in both groups. Haemoglobin (Hb), iron status and inflammation were assessed every two weeks. Fractional iron absorption at each time point and cumulative absorption was quantified by measuring erythrocyte incorporation of ^57^Fe and compared using mixed models.

**Results:**

Comparing group A and B, geometric mean iron absorption did not differ on the first day of supplementation (9.0% *vs*. 11.4%, *P* = 0.213) and cumulative iron absorption from the three time points did not differ (6.0% vs. 7.2%, *P* = 0.124). Hb concentration increased in both groups two weeks after malaria treatment (*P* < 0.001) and did not differ after eight weeks of supplementation (*P* = 0.542).

**Conclusions:**

In anaemic toddlers after uncomplicated malaria, a two-week delay in starting iron supplementation did not significantly increase iron absorption or Hb concentration. Iron absorption is sufficiently high in the immediate post-malaria period to warrant supplementation. These findings suggest there is no need to change the current practice of immediate iron supplementation in this setting.

**Trial registration:**

This trial was registered at Pan African Clinical Trials Registry (pactr.org) as PACTR2010050002141682.

## Introduction

Anaemia affects more than 1.7 billion people worldwide [1]. In low-income countries of sub-Saharan Africa, major causes of anaemia are infectious diseases, particularly malaria, and diets low in bioavailable iron [2, 3]. Malarial anaemia is caused by a combination of red cell lysis and inflammation. During malaria, high levels of pro‒inflammatory cytokines increase serum hepcidin and this limits the release of recycled red cell iron sequestered in macrophages and other reticuloendothelial cells [4] and reduces dietary iron absorption [5, 6]. Both mechanisms decrease the amount of circulating iron resulting in impaired erythropoiesis and the anaemia of inflammation [7]. Therefore, in a child presenting with malaria and anaemia, the anaemia may be due to red cell lysis, nutritional iron deficiency and inflammation caused by the infection.

The prevalence of iron deficiency among Malawian pre-school age children in the rural region is high, about 75%, and similar rates are reported from other African countries [8]. In sub-Saharan Africa, a child with malaria and anaemia is often given anti-malarial treatment and iron supplementation [9], and the iron supplements are usually started directly after the malarial treatment, in order to reduce clinic visits and increase compliance. Although there are concerns that iron supplements may increase the intensity of a *Plasmodium* infection [10], they are recommended for anaemic children in areas of adequate malarial surveillance and health care [9]. However, the efficacy of providing iron immediately after malarial treatment has been questioned. Doherty *et al.* supplemented young Gambian children aged 18 to 36 months with daily 2 mg/kg elemental iron (as iron glycine sulphate) directly after treatment of acute uncomplicated malaria. Using stable iron isotopes, they found that iron absorption was 8% when given directly after the treatment and was 15% after 2 weeks; the authors hypothesized this was due to lower inflammation at the later time point [5]. Afebrile (asymptomatic) *Plasmodium falciparum* malaria causes inflammation which decreases iron absorption in Beninese women by 40% [6] and in Ivorian children by 100% (Glinz *et al*., personal communication). These and the findings from Doherty suggest that iron absorption within the first two weeks after treatment of acute malaria is not sufficient to warrant iron supplementation in this period. Hence, iron supplements begun two weeks after treatment of acute malaria might be twice as well absorbed than those given immediately after treatment.

Therefore, the objective of the present study was to measure iron absorption from iron supplements at three subsequent time points in toddlers with acute uncomplicated malaria, who were treated against malaria and had begun iron supplementation either immediately after treatment (group A) or after a delay of two weeks (group B). In the group A, iron absorption was measured at 0, 2 and 8 weeks after anti-malarial treatment, and in group B, at 2, 4 and 10 weeks after anti-malarial treatment. The hypothesis of the present study was that cumulative iron absorption at the three time points would be greater in group B than in group A.

## Methods

### Study site and study population

The study was conducted in southern Malawi at the Zomba Central Hospital in Zomba, Malawi. The study was initiated in January 2012 and was completed in September 2013. The present study joined forces with a larger randomized controlled trial, which is assessing adverse events of iron supplementation in children after anti-malarial treatment and is aiming to assign 600 children. The present study was conducted in parallel of this study, i.e. children were either assigned to the larger trial or to the present study, but benefitted from the infrastructure (trained technicians, nurses, etc.). Malaria transmission is moderate-to-intense perennial with a seasonal peak, i.e. large rainy season from November to March. A sample size of 18 children in each group was calculated to be sufficient to detect a 40% difference in iron absorption between study groups, based on a standard deviation of 0.228 in (log-transformed) iron absorption from previous studies at the ETH Zurich [6, 11, 12], and 80% power and a type I error rate of 5%. Anticipating a 10% drop-out rate, 20 children in each study groups was deemed to be satisfactory.

Toddlers were recruited at Zomba Central Hospital if they met the following inclusion criteria: i) aged 12–24 months; ii) body weight 8–12 kg; iii) moderate anaemia defined as Hb concentration between 70–100 g/L; iv) documented uncomplicated malaria and completed treatment with artemether-lumefantrine dispersible (LA), [artemisinin (20 mg) and lumefantrine (120 mg), Novartis, Basel, Switzerland, per day, over three consecutive days]; v) history of fever within the last 48 hours; vi) no known adverse reactions to oral iron; vii) HIV sero-negative; viii) absence of major systemic illnesses; ix) anticipated residence in the study area for the entire study duration; x) no iron supplementation in the month preceding enrolment; xi) written informed consent from a parent/guardian to participate in the study. Documented uncomplicated malaria was referred to when it was documented in the health passport or other hospital notes on the out-patient department for a child who presented to the hospital with a) history of fever in the previous 24 hours or was febrile on the examination with or without other symptoms of malaria like general body pains; and b) was positive for *P. falciparum* by microscopic examination of the blood smear. All signs of severe malaria as defined by WHO of severe anaemia, respiratory distress or altered consciousness were excluded for on examination [13]. In sequence, toddlers fulfilling these criteria were alternately assigned into the two groups. Reasons for discontinuation of the study were: i) problems with the administration of the stable iron isotope, ii) repeated difficulties with the blood taking, iii) any infection or injuries leading to hospitalization, or iv) recurrent *P. falciparum* infection that became febrile. If a child dropped out of the study, another child was recruited into that group to reach a number of 20 children completing the study in each group (i.e. 19 children in group A completed visit 6 and 20 children in group B completed visit 7).

### Study procedures

**Figure 1:**
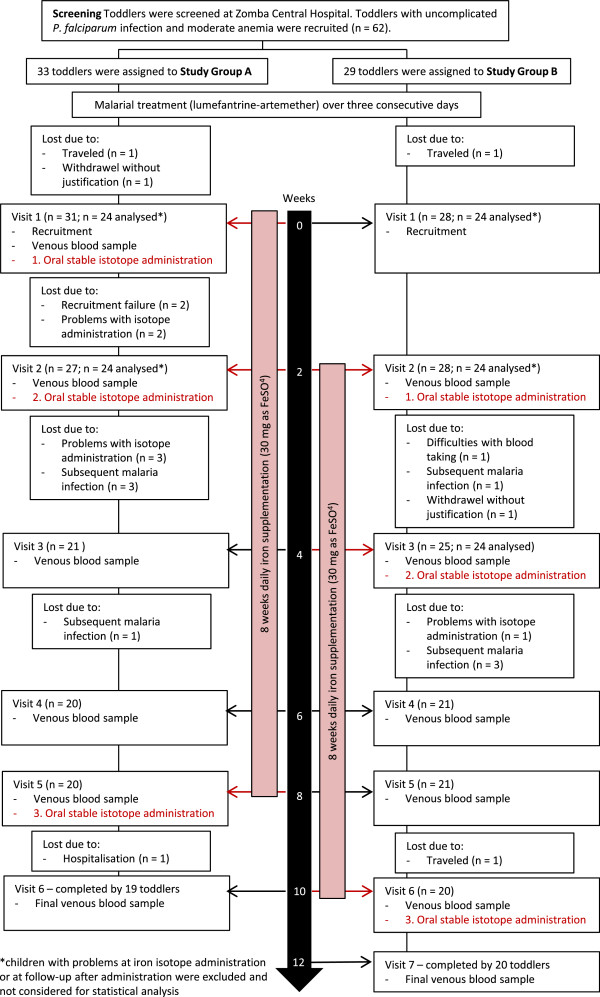
**Study flowchart.** Overview of the study design of the stable iron isotope absorption studies conduction in Malawian toddlers aged 12 to 24 months.

The study started on the first day (visit 1) after completion of the anti-malarial treatment with LA. All children received daily iron supplementation of 30 mg iron (assuming an average body weight of 10 kg) for a period of eight weeks. During this period, a total of three iron absorption studies in each group was conducted. Children’s full medical history was recorded, the children were examined on the day of recruitment (visit 1) and demographic information were collected including a detailed location map of their residence. Participants in study group A started with iron supplementation on the first day after completion of malarial treatment (visit 1). In group A, the time points for iron absorption studies were at the first day after anti-malarial treatment completion (visit 1), after 2 weeks (visit 2) and after 8 weeks (visit 5) (Figure 1). Participants in study group B started with iron supplementation two weeks after they had completed their malarial treatment. The time points for the iron absorption studies in group B were at 2 weeks (visit 2), 4 weeks (visit 3) and 10 weeks (visit 6) after the completion of malarial treatment (Figure 1). Each mother was asked to come for a scheduled follow-up visit at the research unit at the Zomba Central Hospital every other week.

### Iron supplementation and iron isotope preparation and administration

Children received iron sulfate (30 mg Fe daily, assuming a mean body weight of 10 kg) as syrup (Enfamil® Fer-In-Sol®, Mead Johnson & Company, USA) for eight weeks, except on the three days of the stable iron isotope studies. The mothers were trained in the iron supplementation administration and the handling of the dispenser. Mothers administered the iron directly into the mouth of the child from a dropper. The bottles containing iron syrup were weighted before bottles were handed out to the mothers and weights were recorded in a log-file. Mothers were asked to bring back the bottles for each visit, when the bottles were weighted to ascertain compliance and if needed mothers were advised with respect to correct administration.

The stable iron isotope (elemental ^57^Fe, 97.82% isotopic enrichment) were obtained from Chemgas, Boulogne, France. ^57^FeSO_4_ was prepared from isotopic enriched ^57^Fe by dissolution in 0.1 mol H_2_SO_4_/L. On the day of stable isotope administration, children received an oral dose of 27 mg unlabelled Fe and 3 mg ^57^Fe (both as FeSO_4_ in solution) in the morning after at least a two-hour supervised fast. The acidified solution (FeSO_4_) was sweetened by adding 0.5 ml orange squash (Sobo orange syrup, Southern Bottlers, Blantyre Malawi). The solution was kept refrigerated (5°C) in plastic containers. The same study nurse performed the isotope administration with a disposable dropper (plastic Pasteur pipette) for all participants and the local investigator supervised the administration procedure. The plastic container with iron isotope and dropper were rinsed twice with 2 ml water-orange squash mix (1:1). All children were observed for three hours after iron isotope administration and any spillage or vomiting was recorded. The protocol of iron isotope administration was repeated after two and eight weeks. In between the time points of isotope administration, children continued with normal iron supplementation of 30 mg/day for a total of 8 weeks. Group A finished the supplementation 8 weeks after malarial treatment and the last blood sample was drawn after 10 weeks. Group B finished the supplementation with a two-week delay, i.e. at week 10, and the last blood sample was drawn 12 weeks (visit 7) after treatment.

### Blood analysis

Venous blood samples (3 ml) were collected in EDTA-coated tubes (Becton Dickinson AG, Allschwil, Switzerland) on the day of iron isotope administration (before the administration) and every other week. For group A, the first full blood sample was collected at visit 1 and the last at visit 6. For group B, the first full blood sample was collected at visit 2 and the last at visit 7 (Figure 1). When insufficient blood was obtained from a venepuncture for all analyses, isotopic analyses were prioritized.

The HIV status of the child was examined at recruitment with Determine HIV Test (Alere GmbH, Switzerland). Hb concentration was measured with a HemoCue® Hb 201^+^ (HemoCue AB, Angelholm, Sweden) on the screening day. Subsequently, full blood count was performed with an automated haematology analyzer Sysmex KX-21 N (Sysmex, USA). One ml full blood was frozen at −20°C for iron isotope quantification. Plasma was separated and frozen at −20°C under temperature controlled condition avoiding freeze-thaw cycles. Frozen full blood and plasma samples were transferred to Switzerland and Germany. The α1-acid glycoprotein (AGP), C-reactive protein (CRP), and plasma ferritin (PF) concentrations were analysed in Germany with a sandwich enzyme-linked immunosorbent assay described elsewhere [14]. Although it was planned to measure soluble transferrin receptor, for technical reasons it was not possible to accurately measure it in the collected samples, possibly due to the presence of EDTA in the collection tubes (Jürgen Erhardt, personal communication, 2014).

Isotope enrichment was quantified in the Laboratory of Human Nutrition at ETH Zürich, Switzerland. The enriched whole blood samples were mineralised by microwave digestion and iron was separated by anion-exchange chromatography and a subsequent solvent-solvent extraction step into diethylether [15]. Iron was analysed by negative thermal ionization mass spectrometry (TIMS) with a magnetic sector field mass spectrometer (Finnigan MAT 262; Thermo Finnigan, Bremen, Germany) equipped with a multicollector system for simultaneous ion beam detection. The calculation of amount of ^57^Fe label present in the blood was based on isotope-dilution [15]. Circulating iron was calculated from the blood volume based on height, weight and Hb concentration [16], assuming that the blood volume in children after malarial treatment is not different from healthy children. One hundred percent incorporation of the absorbed iron into red blood cells was assumed for calculation of fractional absorption [17]. The blood collected before each label administration was used as the isotopic enrichment baseline for the subsequent absorption calculation. The cumulative iron absorption was calculated based on the sum of the three oral doses, the isotopic enrichment measured in the very last collected blood sample, and the very first isotopic enrichment baseline sample. Because the calculations of the fractional iron absorption and the calculations of cumulative iron absorption are different, the number of quantified iron absorptions at the last time point and the number of quantified cumulative iron absorptions might be different.

Thick blood smears for *P. falciparum* asexual parasites were prepared and examined. A thick smear was considered as negative if 100 microscopic fields revealed no parasites. Thick blood films were stained with Giemsa and were microscopically examined for *Plasmodium* species parasitaemia. Malaria parasites were counted against 200 leucocytes. Counts were converted to number of parasites/μL blood assuming 8,000 leucocytes/μL blood [18].

### Ethics

The ethics committees of ETH Zürich (reference no. EK 2011-N-54) and College of Medicine, Blantyre (COMREC P.01/10/859) approved the study. The trial was registered at pactr.org (PACTR2010050002141682). Health authorities and parents/guardians of the participating children were informed about the purpose, procedures, and potential risks and benefits of the study. Written informed consent was obtained from parents/guardians of the participating children. Participation was voluntary; hence one could withdraw from the study at any time without further obligations. Participants received no remuneration for participation, but travel costs for scheduled and sick visits were paid by the study.

### Statistics

Data was double entered in Microsoft Access 2010 (2010 Microsoft Corporation) and verified by a data officer against original clinical records. Data was analysed with STATA version 11.1 (StataCorp LP; USA). All children were included into the final analysis who had an iron absorption measurement from first stable iron isotope study (completion of visit 2 in group A and completion of visit 3 in group B), even if they subsequently missed follow-up visits. Iron absorption, iron status and inflammation parameters were assessed with linear regression mixed (random and fixed effects) models. When modelling, both time (visits) and group were considered as categorical and the random effects allowed to vary within participants (child-level). For each time point, the residuals of all parameters were assessed for normality with Q-Q and box plots. If residuals were not normally distributed, data were log-transformed. All models were assessed for time effects within groups and interaction of study groups and time. The geometric mean iron absorption of all isotope doses (total isotopic enrichment) in the last collected blood sample was compared between both groups with a simple t-test for unpaired data, after the individual data was log transformed.

## Results

### Subjects

Forty-eight children completed the first iron absorption study (24 each in group A and B) and were considered for statistical analysis with mixed models. Twenty-three children dropped out during the study (Figure 1). Twenty (87%) of the drop-outs occurred within the first four weeks (i.e. up to the third visit).

The mean (±SD) age at visit 1 was 19.0 ± 3.9 months in group A and 16.7 ± 3.8 months in group B. Fifty percent of the children in group A and 58% in group B were boys. Mean (±SD) body weight at baseline was 10.2 ± 1.0 kg in group A and 9.8 ± 1.0 kg in group B. Of the children in group A, 21, 15, and 14 subjects completed the first, second and third iron absorption studies respectively. In group B, 20, 16 and 16 subjects completed the first, second and third iron absorption studies respectively. Seventeen children in group A and 16 children in group B received the iron isotopes at all 3 time points and provided the final blood sample, and for these the cumulative isotopic enrichment over the eight-week study was calculated.

### Treatment of malaria and reinfection

All toddlers had no malaria parasites on the day after completing malarial treatment. Re-infection was common: at visit 2 (two weeks after treatment), one child in both groups was re-infected; at visit 3 (four weeks), one child in group B was re-infected; at visit 4 (six weeks) one child in group A and three in group B were re-infected; at visit 5 (eight weeks), one child in group A was re-infected; at visit 6 (10 weeks) no child was re-infected and at visit 7 (12 weeks) two children in group B were re-infected.

### Iron absorption

**Figure 2:**
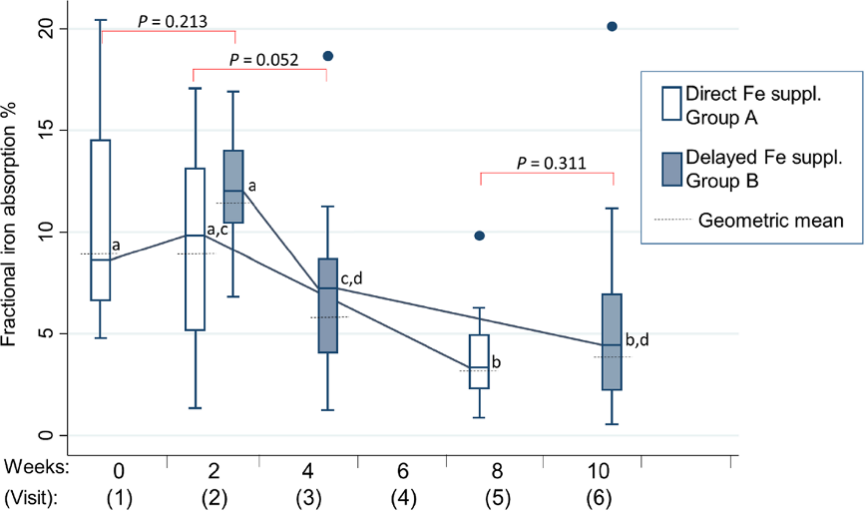
**Box plots of iron absorption after uncomplicated malaria.** Box plots of fractional iron absorption at three time points during iron supplementation measured in Malawian toddlers assigned to group A (n = 24) and group B (n = 24). Each boxplot represents the medians, 25^th^ and 75^th^ percentiles, lower and upper limits, and dots represent outliers. Additionally, the geometric mean was added as dashed line. Toddlers in group A started the 8-week iron supplementation immediately after malarial treatment and the iron absorption was quantified at week 0, 2 and 8. Toddlers in group B started the 8-week iron supplementation with a 2-week delay and iron absorption was measured at week 2, 4 and 10. Abbreviations: Fe, iron. ^a,b,c,d^Within and between group comparison: different letters indicate statistical significance (*P* < 0.05), i.e. if two mean/median values within one parameter carry the same superscript letter, they are not significantly different, i.e. *P* > 0.05.

Geometric mean fractional iron absorptions at visit 1, visit 2 (two weeks) and visit 5 (eight weeks) in group A were 9.0% (95% CI: 7.4 – 11.0%), 8.8% (95% CI: 6.5 – 11.8%) and 3.1% (95% CI: 2.1 – 4.7%) (Figure 2). In group B, geometric mean iron absorptions at visit 2 (two weeks), visit 3 (four weeks) and visit 6 (10 weeks) were 11.5% (95% CI: 10.0 – 13.1%), 5.7% (95% CI: 3.9 – 8.5%) and 3.9% (95% CI: 2.4 – 6.4%). Fractional iron absorption was not significantly different (*P* = 0.213) between group A (visit 1) and group B (visit 2). Mean fractional iron absorption was also not significantly different between the groups at two weeks (*P* = 0.052) and eight weeks (*P* = 0.311) The geometric mean fractional iron absorption (measured by cumulative isotopic enrichment) from the three isotope doses over the eight-week period was 6.0% (95% CI: 5.1 – 7.1%) in group A and 7.2% (95% CI: 6.1 – 8.4%) in group B, and was not significantly different (*P* = 0.124). Iron absorption in group A did not change between visit 1 and visit 2, but decreased significantly from 8.8% at visit 2 to 3.1% at visit 5 (*P* = 0.002). Iron absorption in group B decreased significantly from 11.8% at visit 2 to 5.9% at visit 3 (*P* = 0.001), but did not differ between visit 3 and visit 6.

### Inflammation and iron indices

In group A, median CRP and AGP concentrations were elevated directly after completion of malarial treatment (Table 1); two weeks later, the inflammation had resolved. No blood samples from group B at visit 1 were collected; the first venous blood sample in group B was collected at visit 2, i.e. two weeks after anti-malarial treatment completion, and inflammation status was not different from group A at visit 2. From two weeks onward, inflammation markers remained normal in both groups.

**Table 1:** **Iron absorption, iron and inflammation parameters among Malawian toddlers after anti-malarial treatment**

		1. visit	2. visit	3. visit	4. visit	5. visit	6. visit	7. visit
		0 weeks	2 weeks	4 weeks	6 weeks	8 weeks	10 weeks	12 weeks
**Absorption (geometric mean%, 95% CI)**								
	A	9.0^a^ (7.4-11.0)	8.8^a,c^ (6.5-11.8)	N.A.	N.A.	3.1^b^ (2.1-4.7)	N.A.	N.A.
	B	N.A.	11.5^a^ (10.0-13.1)	5.7^c,d^ (3.9-8.5)	N.A.	N.A.	3.9^b,d^ (2.4-6.4)	N.A.
**Haemoglobin concentration (g/L, mean, ±SD)**								
	A	84^a^ ± 7	104^b^ ± 9	113^d^ ± 10	116^d^ ± 9	111^c,d^ ± 12	114^d^ ± 13	N.A.
	B*	80^a^ ± 9	100^b^ ± 9	106^c^ ± 12	108^c^ ± 11	109^c,d^ ± 13	114^d^ ± 14	111^c,d^ ± 12
**Plasma ferritin (μg/L, median, 25th 75th)**								
	A	189^a^ (142–263)	57^b,c,d^ (36–94)	56^b,c,d^ (38–84)	49^b,c,d^ (40–90)	49^b,c^ (34–64)	45^b,c^ (30–69)	N.A.
	B	N.A.	39^c^ (14–52)	46^c,b^ (28–102)	57^b,d^ (34–88)	66^b,d^ (37–99)	77^b,d^ (45–169)	102^d^ (56–163)
**C-reactive protein (mg/L, median, 25th 75th)**								
	A	36.7^a^ (24.2-44.8)	1.6^b^ (0.5-3.3)	3.6^b^ (0.5-10.3)	1.6^b^ (0.8-5.1)	2.1^b^ (0.8-9.1)	1.4^b^ (0.7-5.0)	N.A.
	B	N.A.	2.5^b^ (1.3-7.3)	2.2^b^ (0.6-4.4)	4.3^b^ (1.8-10.9)	3.3^b^ (0.8-7.9)	1.1^b^ (0.6-5.9)	4.3^b^ (2.9-8.5)
**AGP (g/L, median, 25th 75th)**								
	A	1.86^a^ (1.56-1.98)	1.05^b^ (0.88-1.52)	0.98^b^ (0.88-1.27)	1.07^b^ (0.69-1.23)	1.09^b^ (0.83-1.38)	1.02^b^ (0.66-1.25)	N.A.
	B	N.A.	1.25^b^ (0.96-1.55)	1.06^b^,c (0.70-1.37)	1.07^b^,c(0.84-1.6)	1.01^b,c^ (0.80-1.37)	0.86^c^ (0.69-1.14)	1.10^b,c^ (0.89-1.33)

Malawian toddlers presenting with post-malarial anaemia were supplemented with 30 mg iron for 8 weeks, either directly after malaria treatment (group A, n = 24) or starting with a 2-week delay (group B, n = 24). Children received a dose of a stable iron isotope (^57^Fe) at three occasions, i.e. group A at week 0, 2 and 8 and group B at week 2, 4 and 10. Iron and inflammation parameters are stratified by iron supplementation group and visits. Abbreviations: AGP, α1-acid glycoprotein; CI, confidence interval; N.A., not applicable; SD, standard deviation.

^a,b,c,d^If two mean/median values within one parameter carry the same superscript letter, they are not significantly different, i.e. P > 0.05. Different letters indicate significant differences (P < 0.05) within and between groups A and B for iron absorption, iron and inflammation parameters were assessed with random effect models.

*Haemoglobin concentration measured with HemoCue 201 in group B at visit 1.

**Figure 3:**
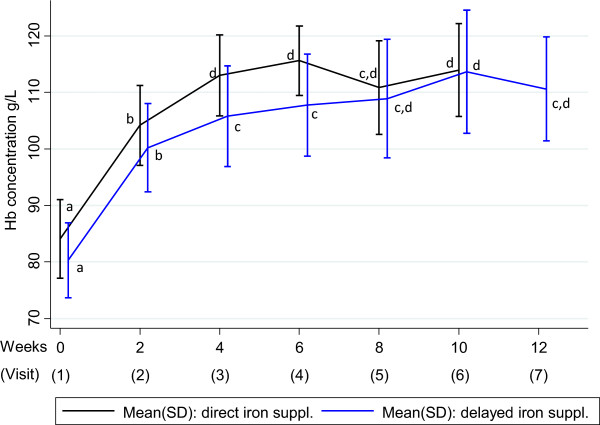
**Recovery of haemoglobin concentration after uncomplicated malaria.** Haemoglobin (Hb) concentration (±standard deviation (SD) indicated by vertical bars) in Malawian toddlers (aged 12 to 24 months) with post-malarial anaemia starting 8-week iron supplementation either directly after malarial treatment at week 0 (black) or starting delayed at week 2 (blue). ^a,b,c,d^Different letters indicate significant (*P* = 0.05) differences between and within groups, i.e. if two mean/median values within one parameter carry the same superscript letter, they are not significantly different, i.e. *P* > 0.05.

Median plasma ferritin (PF) was high directly after malarial treatment in group A and significantly decreased from 189 μg/mL to 57 μg/mL (*P* < 0.001) two weeks later (visit 2) (Table 1). The PF concentration at baseline in Group A was significantly increased compared to the other time points within group A. In the following visits, PF concentration was not different within each group and between the two study groups, except at visit 2 when the PF concentration in group B was significantly lower and at visit 7 in group B when PF concentration increased to 102 μg/mL. At visit 1, mean Hb concentration was 84 g/L in group A and 80 g/L in group B. Mean Hb concentration increased significantly (within group) in both groups A and B two weeks after treatment (at visit 2) to 104 g/L (*P* < 0.001) and 100 g/L (P < 0.001) respectively, and increased further to 114 g/L in both groups during the course of the treatment without statistical difference between the two groups (Figure 3).

## Discussion

The main findings of this study are: a) toddlers receiving iron supplementation with a two weeks delay did not absorb significantly more iron over the eight week supplementation period than the group starting supplementation immediately after treatment; b) in post-malarial anaemia, the first dose of iron supplementation is not better absorbed when given two weeks after malaria treatment than directly after treatment; and c) Hb concentration increased during the first two weeks after malaria treatment, with and without iron supplementation.

The present study did not confirm the findings of Doherty *et al.* in children with acute malaria that iron absorption increased by some 75% (from 8% to 14%) two weeks after malarial treatment compared to iron absorption immediately after malaria treatment [5]. Fractional iron absorption in our children in group A immediately after malarial treatment and two weeks later was similar, although the absorption at visit 1 in the group A receiving supplements immediately was 27% lower than in group B (*P* = 0.213) receiving supplements after a 2 week delay at visit 2. One possible explanation for the different outcomes in the Gambian study and our study is that the 18–36 month old Gambian children received 3.9 mg ^57^Fe as isotopically labelled ferrous sulphate in orange juice on the day after the malarial treatment but a lower amount of iron (1.3 mg ^58^Fe) at the second absorption study 2 weeks later [5]. Fractional iron absorption is influenced by the amount of iron administered [19] and is lower with higher amounts of iron. Additionally, the Gambian children were administered isotopes with orange juice, which would be expected to contain ascorbic acid which increases iron absorption [20] and might have a stronger influence on iron absorption in Gambian children without infection than those with infection.

However, other studies support the observations of Doherty *et al.* from the Gambia. These include studies from Benin which reported that iron absorption from an iron fortified sorghum porridge by Beninese women with malarial parasitaemia increased after malarial treatment [6] and our studies in Ivorian children with malarial parasitaemia (Glinz *et al*., personal communication) which found that iron absorption from an iron supplement was increased by 40%-100% two weeks after malarial treatment. In both studies, inflammation parameters were increased during afebrile malaria but normalized two weeks after malarial treatment [6].

Immediately after malarial treatment, the inflammation indicators CRP and AGP in group A were elevated, but they decreased significantly within 2 weeks to a level which then remained constant over the following weeks. This plateau level in group A was similar to the level in group B, which also remained relatively constant over the supplementation period. However, although inflammation biomarkers decreased in group A, as discussed above, fractional iron absorption did not increase over this two week period. This might be explained by a lag-time in the response of hepcidin compared to the inflammation biomarkers, or be related to the daily supplementation of 30 mg iron and improved iron status leading to a lower iron absorption. Iron absorption in group B decreased significantly over the first 2 weeks of iron supplementation.

The results of the present study suggest that a fairly high percentage of iron (9.0%) was absorbed when the supplements were given immediately after anti-malarial treatment. This amount of absorbed iron is likely to be important to an anaemic child during early recovery from malarial anaemia. This finding supports the current practice of immediate iron supplementation of anaemic or iron deficient children.

Based on the Hb concentration at the end of supplementation, there appears to be no advantage of delaying supplementation by two weeks. Hb concentration increased within two weeks of malarial treatment irrespective of whether the child received iron supplements or not. This is similar to the Gambian study [5] where Hb concentration after malarial treatment in the anaemic children increased at a faster rate than in anaemic children with iron deficiency but without precedent malaria episode. This suggests that erythropoiesis was suppressed due to malaria and recycled red cell iron was trapped in the reticuloendothelial macrophages [7, 21]. In addition to impaired erythropoiesis due to the lack of iron, it is also possible that erythropoiesis was impaired by pro- and anti-inflammatory factors [22–24] or by the malaria pigment haemozoin [25], that are increased during malarial infection. After malarial treatment, it appears that normal erythropoietic activity is re-established by mobilization of sequestered iron from macrophages in the reticuloendothelial system [5]. Because recycled red cell iron provides >90% of the iron for erythopoeisis, while only 5-10% comes from absorbed dietary iron, iron release from the macrophages after the malarial treatment was probably responsible for the rapid increase in Hb concentration in both groups. The findings of the present study are in contrast to an earlier study among Malawian children which reported that Hb concentration increased significantly after anti-malarial treatment in children receiving iron supplements during four weeks compared to children receiving no iron [26]. However, these studies have a limited comparability, because group B in our study was not a control group.

The present study has several limitations. It was aimed to complete the study with approximately 20 children in each study group. Because of a drop-out of n = 14 (42%) in group A and n = 9 (31%) in group B it cannot be ruled out that this created the risk of potential bias. However, the reasons for drop-out were diverse and similar in both groups and probably did not create a risk of bias. Moreover, the inclusion criteria were narrow and further reduced the risk of bias. Another study limitation is the alternate assignment to study group A and B, which is not considered as proper randomization method and hence, alternate assignment harbours the risk of potential imbalances between group A and B at recruitment. Moreover, no venous blood samples were collected from group B at the first visit, thus potential imbalances between group A and B cannot be assessed. A further study limitation might be the diminished iron absorption due to recurrent *P. falciparum* infection, which remained asymptomatic, i.e. 12% in group A and 14% in group B. However, as asymptomatic recurrent infections occurred to a similar extent in both groups, iron absorption was probably influenced in a similar way, although the recurrent infections might have reduced the contrast between both groups.

The present study did not address the potential safety concerns of iron supplementation in young children in sub-Saharan Africa. Iron may increase the intensity of malarial infections and other infectious diseases [27, 28]. Also, unabsorbed iron may increase the pathogenicity of the gut microbiota [29], and the incidence of diarrhoea [30, 31]. WHO recommends supplementation when it is accompanied by malarial surveillance and good health care [9]. The participating children had no longer a detectable *P. falciparum* infection after the full course of treatment with LA and were free of malaria when supplementation started. However, the half-life of artemisinin is only 2–3 hours, and the half-life for lumefantrine is about 3–4 days [32]. These short half-lives provide protection against re-infection for only two to three weeks [33]. As the study period was eight weeks, there was an issue of safety [9], so the children were continuously monitored throughout the eight weeks supplementation period, plus two weeks after completion of iron supplementation, and did detect and treat reinfection. Hence, close medical supervision was provided after the protective effect of anti-malarial therapy had faded. Children with asymptomatic parasitaemia were continued in the study, but children with malaria (i.e. febrile *P. falciparum* infection) were withdrawn. The risks of iron supplementation after anti-malarial treatment were not assessed in the present study. This issue is being evaluated in our larger ongoing, randomized control trial, which is assessing the safety of iron supplementation after malarial treatment among 600 children. The strengths of our present study are the longitudinal design, repeated measurement of iron absorption using stable isotopes, and the biweekly follow-up in Malawian toddlers.

## Conclusion

In conclusion, in anaemic toddlers after uncomplicated malaria, a two-week delay in starting iron supplementation had no measurable benefit on iron absorption or on the recovery of Hb concentration. The findings of the present study show that iron absorption is sufficiently high immediately after anti-malarial treatment and hence, these findings suggest that there is no need to change the current practice of immediate iron supplementation, considering that compliance from supplementation given immediately after malaria treatment is generally thought to be higher in this setting.

## Abbreviations

AGP: α1-acid glycoprotein
CRP: C-reactive protein
Hb: Haemoglobin
PF: Plasma ferritin.
